# Simple urine test might predict heart failure risk: Dutch study

**Published:** 2023

People with consistently high levels of urinary albumin excretion (UAE) and serum creatinine in their urine are at higher risk of developing heart failure, according to recent research, with the study team saying this supports the known connection between renal failure and heart failure. The scientists, from the University of Groningen in The Netherlands, had analysed urine-sample data from nearly 7 000 Dutch participants who were 28 to 75 years old at the start of the study, which followed them for 11 years.

Medical News Today reports that the results found people with stable and high levels of both UAE and serum creatinine in their samples had a higher risk of experiencing heart failure for the first time, while those with elevated levels of UAE had an increased risk of dying from all causes. Similarly, high levels of serum creatinine were not found to be linked to all-cause mortality.

The study, published in the European Journal of Heart Failure, was an attempt to explore the potential health risks for people whose UAE and serum creatinine levels remain high over the long term instead of fluctuating, as they do in most people. The findings may provide physicians with a new biomarker of susceptibility for heart failure, said the authors.

‘Circulating in the bloodstream are lots of substances, some very tiny, for example, sodium or glucose molecules, and some large, such as proteins and antibodies,’ said Dr Richard Wright, a cardiologist specialising in heart failure and transplantation cardiology at Providence Saint John’s Health Centre, who was not involved in the study.

An important function of the kidneys is to filter extra fluid and waste, including acids produced by cells. When they are functioning properly, the kidneys help maintain a healthy balance of chemicals in the blood.

Wright said albumin is the most common protein circulating in the bloodstream. ‘As a large molecule, the filter of the kidney normally does not allow albumin to appear in the urine because it’s too large to make it through the filter. Smaller molecules, such as sugar, pass through to the urine easily.’

As the health of kidneys and their filtration degrade, albumin passes into the urine. This makes its presence there a valuable marker of kidney dysfunction.

‘Serum creatinine is a waste product of muscle use, and found in the blood. It is filtered out of the blood by the kidneys,’ said Dr Jayne Morgan, cardiologist and clinical director of the Covid Task Force at Piedmont Healthcare Corporation, who was also not involved in the study. Higher levels of serum creatinine in the urine are often a sign of declining kidney function, though there are some exceptions. Wright said that for example, weightlifters consume unusually high amounts of protein, so high levels of serum creatinine in their urine do not necessarily signify kidney dysfunction.

He added there was some discussion in the heart failure community regarding older patients who typically have little muscle mass. ‘Creatinine may not be as good a reflection of their kidney function because the creatinine is a derivative of broken-down protein. And if you don’t eat a lot of protein or have a lot of protein in your body, then the creatinine clearance may be misleading,’ he said.

If the loss of renal function is tied to heart failure as more than a symptom, can the loss be reversed? ‘Not really. Renal function declines steadily with age,’ said Morgan. While this loss is inevitable with time, it was possible to slow it down by about half with appropriate medicines, including ACE inhibitors.

## Does this mean new therapies for heart failure?

‘This study continues to connect the kidney and heart in a cardiorenal loop,’ said Morgan. ‘Early albumin excretion is an opportunity to be alerted to not only developing kidney disease but heart failure risk as well.’

She felt that the study’s findings might affect medications prescribed and medical follow up, ‘providing the opportunity for preventative cardiac care, as opposed to interventional cardiac care’.

Dr Andrew Clark, chair of clinical cardiology and head of the department of Academic Cardiology at Hull York Medical School, who was also not involved in the study, cautioned against basing all patient care on these new findings. ‘The study looks at associations between abnormalities in renal function and outcomes and cannot prove a causative link,’ he said, pointing out a limitation of an observational study.

‘In more-or-less any clinical scenario, worsening renal function is associated with worse outcomes, but that doesn’t mean it is the renal dysfunction causing the problem. Any causative association might be the other way round: heart failure potentially causes proteinuria, (abnormal amounts of protein in the urine),’ he said.

He also noted the link the researchers found between these substances and heart failure ‘might simply arise from the fact that the same precursors cause both outcomes. So, for example, high blood pressure and diabetes both cause renal and heart damage.’

Wright suggested including a simple urine test measuring UAE and serum creatinine during check-ups, a test he suspects few doctors prescribe. ‘It’s an inexpensive, easy-to-do test, and carries a lot of prognostic information.’ 
